# Comparative Effectiveness of Four Biologics for Moderate-to-Severe Plaque Psoriasis: A Network Meta-Analysis

**DOI:** 10.7759/cureus.107459

**Published:** 2026-04-21

**Authors:** Abdullah Abdullah, Linda Polus

**Affiliations:** 1 General Medicine, Royal Free NHS Foundation Trust, Barnet Hospital, London, GBR; 2 Dermatology, Ministry of Health Iraq, Baghdad, IRQ

**Keywords:** bimekizumab, biologics, comparative effectiveness, guselkumab, network meta-analysis, psoriasis, risankizumab, secukinumab

## Abstract

Multiple biologic agents targeting different inflammatory pathways are available for moderate-to-severe plaque psoriasis. While previous network meta-analyses have compared biologics, most were published before the approval of bimekizumab (the newest dual interleukin [IL]-17A/F inhibitor) and lack head-to-head comparisons incorporating this agent alongside other contemporary biologic therapies. We conducted a systematic literature search of PubMed and Clinical Trials to identify phase III randomized controlled trials comparing biologic treatments for moderate-to-severe plaque psoriasis. The primary outcome was achievement of 90% or greater reduction in Psoriasis Area and Severity Index (PASI 90) at week 16. We performed a frequentist random-effects network meta-analysis to estimate odds ratios (OR) with 95% confidence intervals (CI) for all treatment comparisons. From 642 records identified, 11 phase III randomized controlled trials (10 datasets, 6,657 patients) were included, comparing bimekizumab (IL-17A/F inhibitor), secukinumab (IL-17A inhibitor), risankizumab and guselkumab (IL-23p19 inhibitors), adalimumab (tumor necrosis factor-alpha inhibitor), ustekinumab (IL-12/23 inhibitor), and placebo. Compared with placebo, all active treatments showed significant efficacy: bimekizumab (OR 170.04, 95% CI 104.33-277.14), risankizumab (OR 95% CI 59.97-151.74), guselkumab (OR 82.25, 95% CI 51.23-132.03), secukinumab (OR 75.01, 95% CI 45.37-124.02), adalimumab (OR 30.07, 95% CI 19.16-47.18), and ustekinumab (OR 26.68, 95% CI 16.48-43.20). Network heterogeneity was low (I² = 0.0%) for most comparisons. Bimekizumab demonstrated the highest efficacy for achieving PASI 90 at week 16, followed by risankizumab, guselkumab, and secukinumab. All newer biologic agents showed substantial superiority over adalimumab and ustekinumab. These findings can inform treatment selection and guideline development for moderate-to-severe plaque psoriasis.

## Introduction and background

Moderate-to-severe plaque psoriasis is a chronic inflammatory skin condition affecting approximately 2-3% of the world’s population [1], characterized by red, scaly plaques covering more than 10% of the body surface area or involving high-impact areas such as the face, hands, or genitalia. The condition causes substantial impairment in the patient's quality of life, affecting daily activities, mental health, and social functioning [2]. Disease severity is measured using the Psoriasis Area and Severity Index (PASI), with PASI 90 representing a 90% or greater reduction from baseline, considered a benchmark for near-complete skin clearance [3]. During the past 20 years, the therapeutic landscape for psoriasis has undergone a transformation with the introduction of biologic agents that target specific inflammatory mediators [4]. Currently available biologics act on several key inflammatory pathways, including tumor necrosis factor-alpha (TNF-α), interleukin-12/23 (IL-12/23), interleukin-17 (IL-17), and interleukin-23 (IL-23)-cytokines that are signaling proteins driving the chronic inflammation seen in psoriatic skin [4,5].

More recent therapeutic agents, especially those inhibiting the IL-17 [4] and IL-23 [5] pathways, have shown enhanced clinical responses in individual clinical trials when compared to earlier-generation TNF-α inhibitors. Nevertheless, direct comparative evidence between all currently available therapies remains limited. Bimekizumab represents the most recently approved agent in this class, receiving regulatory authorization in multiple regions between 2021 and 2023. This drug uniquely targets both IL-17A and IL-17F, distinguishing it from secukinumab, which selectively inhibits IL-17A. Meanwhile, selective IL-23p19 inhibitors such as risankizumab and guselkumab offer the practical advantage of less frequent dosing while maintaining robust clinical efficacy.

Prior systematic reviews and network meta-analyses, a statistical method that allows simultaneous comparison of multiple treatments by combining direct and indirect trial evidence, have attempted to compare the effectiveness of various biologic therapies for psoriasis [6,7]. Important examples include the comprehensive Cochrane review by Sbidian and colleagues published in 2021 [6], which evaluated multiple biologic agents but preceded the publication of bimekizumab’s pivotal phase 3 trial results. Similarly, Armstrong and coworkers conducted a meta-analysis in 2020 [7] that included earlier-generation biologics. However, these previous analyses share several important limitations. First, they were conducted before the pivotal bimekizumab phase 3 trials (BE SURE [8], BE RADIANT [9], BE VIVID [10], BE READY [11]) were published. Second, none provide network comparisons incorporating bimekizumab alongside established therapies. Third, recently published head-to-head trials, specifically IMMerge [12], which directly compared risankizumab versus secukinumab, and BE RADIANT [9], which directly compared bimekizumab versus secukinumab, now provide valuable direct evidence that can strengthen network meta-analysis estimates.

The introduction of bimekizumab into clinical practice raises several clinically important questions. What is the comparative effectiveness of dual IL-17A/F inhibition versus selective IL-17A inhibition using secukinumab? Does bimekizumab provide meaningful therapeutic advantages compared to risankizumab and guselkumab? When these agents are analyzed within a comprehensive network, what is the updated hierarchy of treatment efficacy? These questions carry significant implications for developing evidence-based treatment algorithms, making formulary decisions, and guiding day-to-day clinical practice.

Objectives

We undertook a systematic review and network meta-analysis with three primary aims. First, we aimed to compare the relative efficacy of bimekizumab, secukinumab, risankizumab, and guselkumab against established biologic therapies (adalimumab and ustekinumab) for treating moderate-to-severe plaque psoriasis, with particular emphasis on incorporating recently published bimekizumab trial data. Second, we sought to establish a current hierarchy of treatment efficacy based on the achievement of a PASI 90 response at the standardized 16-week timepoint. Third, we evaluated the consistency of evidence throughout the treatment network, taking advantage of newly available direct head-to-head trial comparisons.

## Review

Methods

Protocol Registration and Reporting Guidelines

This NMA was prospectively registered with PROSPERO (CRD420251148352). The study was conducted in accordance with the Preferred Reporting Items for Systematic Reviews and Meta-Analyses (PRISMA) 2020 statement and PRISMA extension for Network Meta-Analyses (PRISMA-NMA) guidelines [13,14].

Protocol deviations included the following modifications to the original registered protocol. The registered protocol planned to include all IL-17 inhibitors (bimekizumab, ixekizumab, secukinumab, and brodalumab) and IL-23 inhibitors. Following systematic screening, we narrowed the scope to focus on bimekizumab (the most recently approved IL-17 inhibitor with dual IL-17A/F targeting), secukinumab (the established IL-17A inhibitor), and the primary IL-23p19 pathway inhibitors (risankizumab and guselkumab). This focused network was chosen to address the primary research objective of evaluating the comparative effectiveness of the newest generation of biologic therapies, particularly bimekizumab’s position relative to current standard-of-care options. While the IXORA-S trial provides direct evidence comparing ixekizumab with secukinumab, inclusion of ixekizumab and brodalumab would have substantially expanded the network without addressing our core research question regarding the comparative positioning of bimekizumab and IL-23 inhibitors. The registered protocol also planned Surface Under the Cumulative Ranking Curve (SUCRA) calculations; however, we chose to rank treatments by odds ratio versus placebo, which provides a more direct and clinically interpretable measure of treatment effect magnitude. SUCRA probabilities require Bayesian network meta-analysis methods, whereas this study employed frequentist analytical approaches for consistency with standard practice in dermatology comparative effectiveness research. A comparative safety analysis was planned but not conducted due to heterogeneous reporting of adverse events across trials; this topic remains an area for future research.

Search Strategy

A systematic literature search was performed in PubMed and Clinical Trials to identify relevant randomized controlled trials evaluating biologic therapies for moderate-to-severe plaque psoriasis. PubMed (searched 10 October 2025): (psoriasis [MeSH Terms] OR psoriasis [Title/Abstract] OR “plaque psoriasis” [Title/Abstract]) AND (bimekizumab [Title/Abstract] OR secukinumab [Title/Abstract] OR risankizumab [Title/Abstract] OR guselkumab [Title/Abstract]) AND (randomized controlled trial [Publication Type] OR randomized [Title/Abstract] OR randomized [Title/Abstract] OR placebo [Title/Abstract]). Filters applied: randomized controlled trials; humans; last 10 years. Total records retrieved: 259. Clinical Trials (searched 16 October 2025): condition: psoriasis; interventions: bimekizumab OR secukinumab OR risankizumab OR guselkumab; Study type: Interventional studies only. Total records retrieved: 383. Trials identified through Clinical Trials corresponding to studies already retrieved via PubMed were considered duplicates and removed prior to screening.

Study Selection

All records identified from the databases were collated, and duplicates were removed prior to screening. After deduplication, titles and abstracts were screened for relevance. Studies considered potentially eligible underwent a full-text assessment. Study selection was guided by predefined inclusion and exclusion criteria aligned with the objectives of the NMA. Discrepancies in eligibility assessment were resolved through discussion between reviewers.

Inclusion Criteria

Studies were eligible for inclusion if they met all of the following criteria. First, the study design had to be a phase III randomized controlled trial. Second, the population had to consist of adults aged 18 years or older with moderate-to-severe plaque psoriasis. Third, interventions had to include bimekizumab, secukinumab, risankizumab, or guselkumab. Fourth, comparators had to be either placebo or biologic comparators forming part of the predefined treatment network, such as adalimumab or ustekinumab. Fifth, outcomes had to include reported extractable efficacy outcomes relevant to comparative effectiveness, such as PASI [3] response rates at standard timepoints. Sixth, publication type had to be primary efficacy reports of randomized trials. Only one primary publication per trial was included to avoid duplication of patient populations.

Exclusion Criteria

Studies were excluded if they met any of the following criteria. We excluded non-randomized studies, observational studies, real-world studies, registry studies, and pharmacokinetic studies. We also excluded phase I or phase II trials, dose-finding studies, and bioavailability studies. Post-hoc analyses, pooled analyses, subgroup analyses, and long-term or open-label extension studies of previously published trials were excluded. Studies involving non-plaque psoriasis populations were excluded, including those focused on psoriatic arthritis, palmoplantar psoriasis, pustular psoriasis, pediatric populations, or other dermatologic conditions. Trials evaluating comparators outside the predefined treatment network, such as ixekizumab, brodalumab, or etanercept, were excluded, as these did not contribute to network connectivity. Duplicate publications reporting data from the same trial population were excluded, as full-text articles were unavailable for data extraction despite reasonable retrieval efforts.

Data Extraction

Two independent reviewers extracted data systematically using standardized extraction forms. Information captured included study characteristics (first author name, publication year, trial acronym, and clinical trial registration number); patient demographic features (mean age, sex distribution, baseline PASI score, disease duration, and proportion with prior biologic exposure); intervention characteristics (drug name, mechanism of action, dose, and dosing schedule); sample sizes for each treatment arm; and primary outcome data (number of PASI 90 responders and total number of patients randomized to each arm at the 16-week timepoint). Discrepancies were resolved through discussion and consensus. For multi-arm trials comparing three or more treatment groups, we included all relevant pairwise treatment comparisons while appropriately accounting for the statistical correlation between treatment arms sharing a common comparator group.

Quality Assessment

We evaluated risk of bias using the Cochrane Risk of Bias 2 (RoB 2) instrument [15], which systematically assesses five key domains: randomization process, deviations from intended interventions, missing outcome data, measurement of the outcome, and selection of the reported result. Risk of bias assessment was performed independently by two reviewers, with disagreements resolved through discussion. All trials included in our analysis were industry-sponsored phase 3 registration trials that demonstrated low risk of bias across all evaluated domains.

Statistical Analysis

Statistical methods included assessment of heterogeneity and inconsistency [16], publication bias testing [17], and PASI 90 response evaluation [3].

We conducted frequentist random-effects NMA using R statistical software (version 4.3.0) with the netmeta package (version 2.9-0). All statistical analyses were performed in RStudio (version 2023.06.1+524). Our analytical approach assumed a common heterogeneity parameter across all pairwise treatment comparisons within the network, which is standard practice in frequentist NMA and is appropriate given the clinical homogeneity of the included phase III trials. For multi-arm trials such as BE RADIANT, which compared bimekizumab, secukinumab, and placebo simultaneously, all relevant treatment arms were included in the analysis while accounting for the statistical correlation between arms sharing a common comparator group. We used a frequentist framework with a random-effects model to account for potential between-study heterogeneity. The NMA combines direct evidence from head-to-head trials with indirect evidence derived through common comparators, providing effect estimates for all possible treatment comparisons, including those without direct head-to-head trial evidence.

We analyzed the proportion of patients achieving 90% or greater reduction in Psoriasis Area and Severity Index (PASI 90) response at week 16, calculating odds ratios (OR) with corresponding 95% confidence intervals (CI) for all possible treatment comparisons. Odds ratios were selected over risk ratios as they are the standard effect measure in logistic regression-based NMA and provide more stable estimates when event rates vary substantially across treatment arms. All analyses adhered to the intention-to-treat principle with nonresponder imputation for patients with missing outcome data, maintaining consistency with the analytical approaches used in the original trial publications. We constructed network plots to provide visual representation of all direct pairwise comparisons between treatments. We verified network connectivity to ensure that all treatments were linked to the network through at least one evidence pathway, either direct or indirect.

We quantified statistical heterogeneity using both I² and τ² statistics. We interpreted I² values according to standard thresholds: 0-25% indicating low heterogeneity, 25-50% indicating moderate heterogeneity, 50-75% indicating substantial heterogeneity, and values exceeding 75% indicating considerable heterogeneity. We assessed the consistency between direct evidence from head-to-head trials and indirect evidence derived through network connections using the design-by-treatment interaction model [11] and the Separate Indirect from Direct Evidence (SIDE) approach with the back-calculation method. For closed loops within the network where both direct and indirect evidence pathways existed, we systematically compared direct estimates from head-to-head trials against indirect estimates calculated from the network. We calculated inconsistency factors, ratio of ratios (RoR), and corresponding p-values for each comparison to test for significant disagreement between direct and indirect evidence.

We evaluated the potential for publication bias using funnel plot visualization (Figure 4, Appendices) combined with multiple statistical tests, including Egger’s regression test, the Begg-Mazumdar rank correlation test, and the Thompson-Sharp test [12] to detect potential small-study effects. We ranked treatments based on their OR compared to placebo. As noted in the protocol deviations section, SUCRA probabilities were not calculated, as these metrics are not available from the frequentist netmeta package. We constructed a league table displaying OR with 95% CI for all possible pairwise treatment comparisons to facilitate comprehensive comparison of treatment effects across the network.

Sensitivity Analyses

We planned sensitivity analyses, including assessment of results using fixed-effect models as an alternative to random-effects models and systematic exclusion of studies with high risk of bias or small sample sizes (defined as fewer than 200 patients per study). Additionally, we conducted leave-one-out analyses to examine the influence of individual studies on pooled effect estimates.

Results

Study Selection

**Figure 1:**
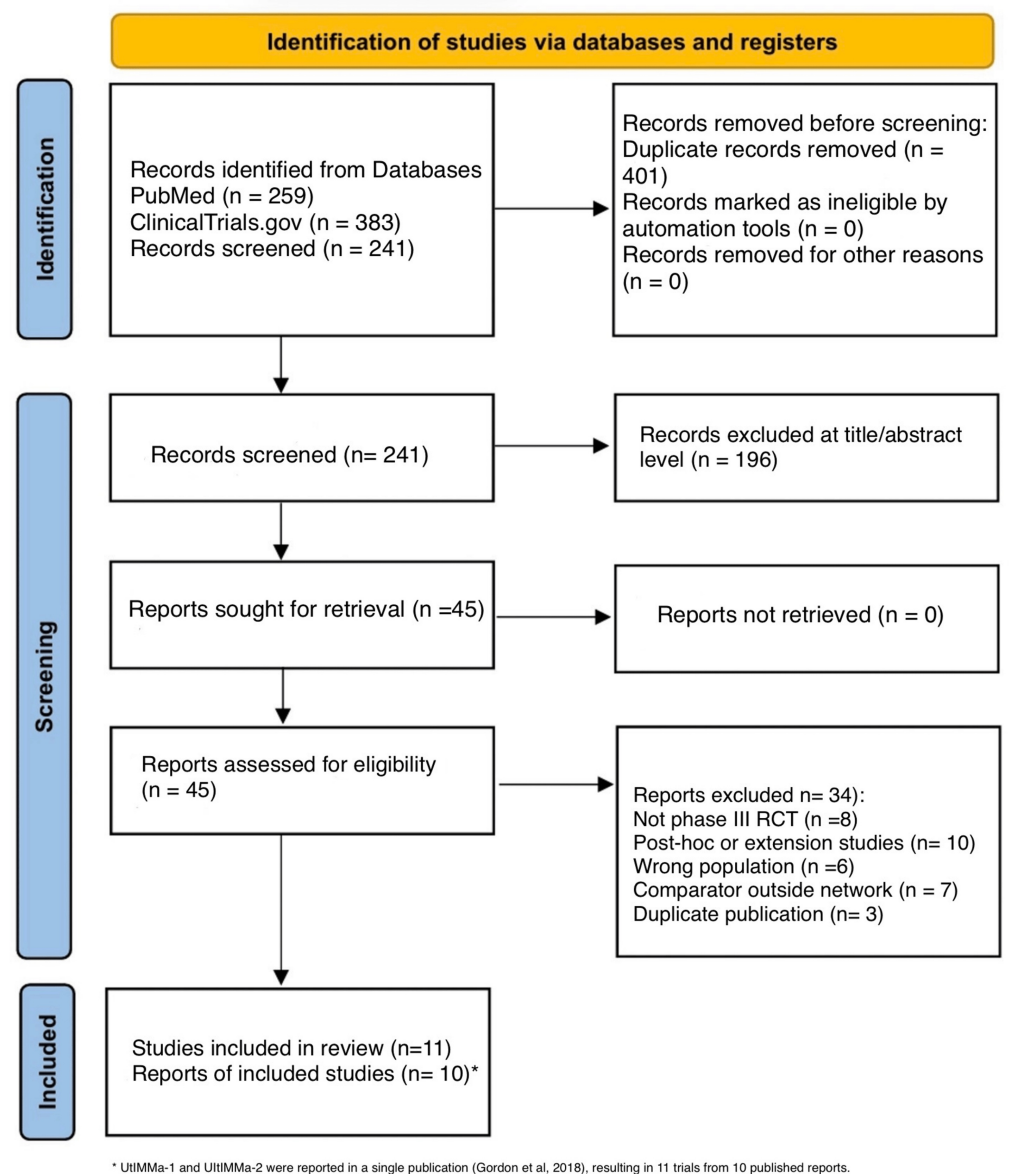
PRISMA 2020 flow diagram

The systematic search identified 642 records (259 from PubMed and 383 from Clinical Trials). After removing 401 duplicates, 241 records were screened by title and abstract, of which 196 were excluded. Forty-five full-text articles were assessed for eligibility. Of these, 34 were excluded for the following reasons: not phase III RCT, post-hoc or extension studies, wrong population, comparator outside network, or duplicate population. Following screening and eligibility assessment, 11 phase III RCTs were included in the final NMA. The UltIMMa-1 and UltIMMa-2 trials were reported as a single pooled dataset in the primary publication by Gordon and colleagues, resulting in 10 distinct study datasets representing a combined total of 6,657 patients. The PRISMA flow diagram is presented in Figure 1.

Characteristics of Included Studies

Table 1 summarizes the characteristics of the 11 included trials [8-22]. These trials constituted the pivotal studies evaluating bimekizumab (BE SURE [8], BE RADIANT [9], BE VIVID [10], and BE READY [11]), secukinumab (BE RADIANT [9] and CLEAR [18]), risankizumab (IMMerge [12], UltIMMa-1 and UltIMMa-2 [21], and IMMvent [22]), and guselkumab (VOYAGE-1 [9] and VOYAGE-2 [20]) and formed a fully connected treatment network suitable for indirect comparison.

**Table 1:** Characteristics of included studies * UltIMMa-1 and UltIMMa-2 were reported together in a single publication by Gordon et al. (2018) [21], resulting in 10 published reports representing 11 individual trials.

Study (Reference)	Study Design	Treatment Arms	Sample Size	Primary Endpoint	Follow-up Duration
BE SURE (Warren 2021) [8]	Phase III RCT	Bimekizumab vs. Placebo	N = 478	PASI 90 at week 16	16 weeks
BE RADIANT (Reich 2021) [9]	Phase III RCT	Bimekizumab vs. Secukinumab vs. Placebo	N = 743	PASI 90 at week 16	16 weeks
BE VIVID (Reich 2021) [10]	Phase III RCT	Bimekizumab vs. Secukinumab	N = 567	PASI 90 at week 16	16 weeks
BE READY (Gordon 2021) [11]	Phase III RCT	Bimekizumab vs. Placebo	N = 435	PASI 90 at week 16	16 weeks
CLEAR (Langley 2015) [18]	Phase III RCT	Secukinumab vs. Placebo	N = 738	PASI 90 at week 16	52 weeks
VOYAGE-1 (Blauvelt 2017) [19]	Phase III RCT	Guselkumab vs. Placebo	N = 837	PASI 90 at week 16	48 weeks
VOYAGE-2 (Reich 2017) [20]	Phase III RCT	Guselkumab vs. Placebo	N = 992	PASI 90 at week 16	48 weeks
IMMerge (Warren 2021) [12]	Phase III RCT	Risankizumab vs. Secukinumab	N = 327	PASI 90 at week 16	52 weeks
UltIMMa-1 (Gordon 2018)* [21]	Phase III RCT	Risankizumab vs. Placebo	N = 506	PASI 90 at week 16	52 weeks
UltIMMa-2 (Gordon 2018)* [21]	Phase III RCT	Risankizumab vs. Placebo	N = 491	PASI 90 at week 16	52 weeks
IMMvent (Reich 2019) [22]	Phase III RCT	Risankizumab vs. Adalimumab	N = 507	PASI 90 at week 16	44 weeks

Patient Characteristics

Across all studies, mean patient age ranged from 43.5 to 48.3 years, with 65-75% male patients. Mean baseline PASI scores ranged from 19.8 to 22.3, indicating severe disease. Prior biologic exposure varied from 13 to 43% across studies. Disease duration ranged from 16.1 to 20.4 years. Table 2 summarizes patient characteristics across included studies.

**Table 2:** Summary of patient characteristics across included studies Ranges represent values across all 11 included trials (10 datasets). BSA: body surface area; PASI: Psoriasis Area and Severity Index.

Characteristic	Range Across Studies	Weighted Mean/Overall
Sample size per study	327 – 992 patients	Total: 6,657
Mean age (years)	42.9 – 54.9	~45
Male sex (%)	63.5 – 74.3	~68
Baseline PASI score	19.0 – 23.1	~21
Prior biologic exposure (%)	13.0 – 41.0	~30
Disease duration (years)	16.1 – 20.4	~18
Mean baseline BSA (%)	23.8 – 33.5	~27
Study completion at week 16 (%)	91.8 – 98.0	~95

Risk of Bias Assessment

All 11 included trials demonstrated low risk of bias across all five domains of the Cochrane RoB 2 tool (Table 5, Appendices).

Network Geometry

**Figure 2:**
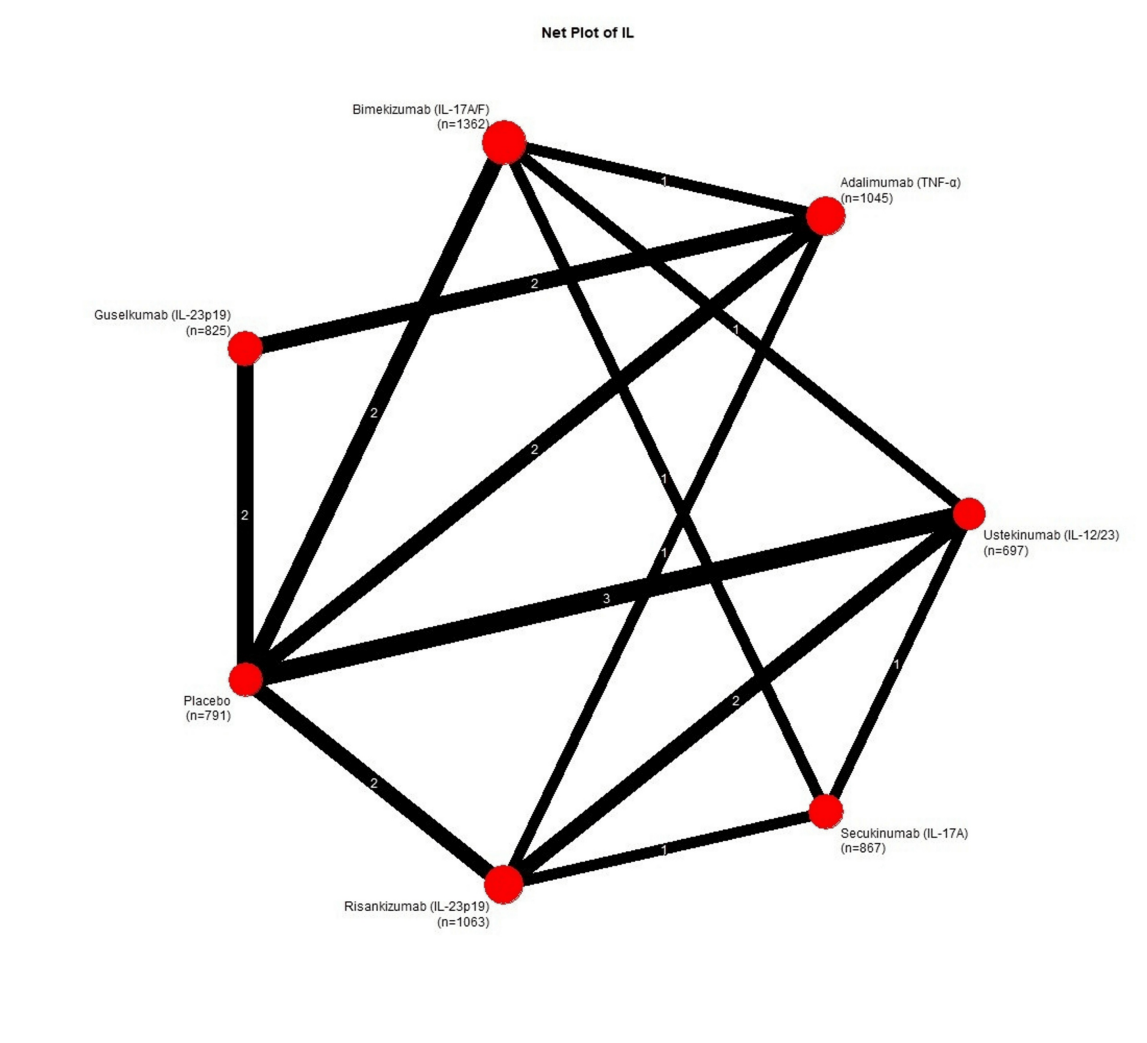
Network plot showing treatment comparisons

The treatment network included seven nodes (six active treatments plus placebo) connected through 13 direct comparisons from 10 studies. All treatments were connected to the network, with placebo and adalimumab serving as common comparators linking multiple treatment pathways. The network plot is presented in Figure 2.

Primary Outcome: PASI 90 Response at Week 16

**Figure 3:**
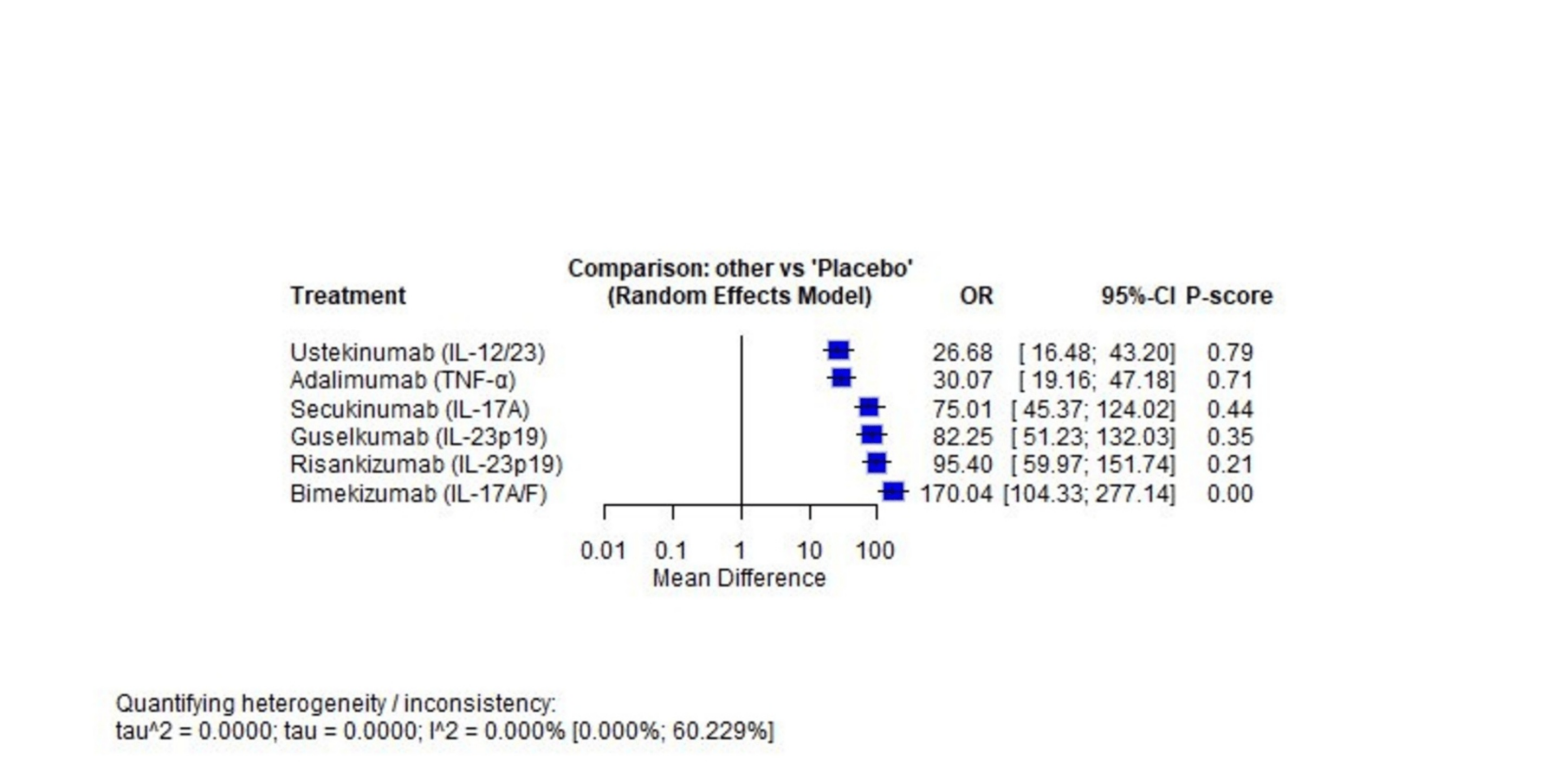
Forest plot of PASI 90 response at week 16 versus placebo

Compared with placebo, all active treatments showed substantial superiority: bimekizumab (OR 170.04, 95% CI 104.33-277.14), risankizumab (OR 95.40, 95% CI 59.97-151.74), guselkumab (OR 82.25, 95% CI 51.23-132.03), secukinumab (OR 75.01, 95% CI 45.37-124.02), adalimumab (OR 30.07, 95% CI 19.16-47.18), and ustekinumab (OR 26.68, 95% CI 16.48-43.20). Figure 3 presents the forest plot showing odds ratios with 95% confidence intervals for PASI 90 response at week 16 compared with placebo.

When compared with adalimumab, the following agents demonstrated superior efficacy: bimekizumab (OR 5.65, 95% CI 4.08-7.84), risankizumab (OR 3.17, 95% CI 2.39-4.21), guselkumab (OR 2.74, 95% CI 2.19-3.42), and secukinumab (OR 2.49, 95% CI 1.75-3.56). Ustekinumab showed no statistically significant difference from adalimumab (OR 0.89, 95% CI 0.63-1.25).

Head-to-Head Comparisons

Bimekizumab demonstrated superior efficacy compared with secukinumab (OR 2.27, 95% CI 1.71-3.01), with direct evidence from BE RADIANT showing OR 2.04 (95% CI 1.41-2.96, I² = 0.58%), demonstrating excellent consistency between direct and network estimates. Bimekizumab also showed superiority versus risankizumab based on network estimates (OR 1.78, 95% CI 1.32-2.41) and versus guselkumab (OR 2.07, 95% CI 1.58-2.71).

Among the IL-23 inhibitors and secukinumab, risankizumab versus secukinumab showed a network OR of 1.27 (95% CI 0.94-1.71), with direct evidence from IMMerge showing an OR of 1.47 (95% CI 0.92-2.37). Risankizumab versus ustekinumab showed a network OR of 3.58 (95% CI 2.74-4.67), with direct evidence from UltIMMa-1 and UltIMMa-2 showing an OR of 3.72 (95% CI 2.66-5.21). Risankizumab versus guselkumab showed OR 1.16 (95% CI 0.81-1.64), indicating no significant difference between these two IL-23p19 inhibitors.

Comprehensive Pairwise Treatment Comparisons

Table 3 presents a league table displaying OR with 95% CI for all possible pairwise treatment comparisons. This table includes both direct evidence from head-to-head trials (shown in bold) and network-derived estimates for treatment pairs without direct comparative trials. Each cell shows the odds ratio and 95% confidence interval comparing the row treatment versus the column treatment. OR greater than 1.0 indicates the row treatment is superior to the column treatment, while OR less than 1.0 indicates the column treatment is superior to the row treatment. Bold values indicate direct evidence from head-to-head randomized controlled trials, whereas regular text indicates network-derived estimates from indirect comparisons. Statistically significant differences occur when the 95% CI does not include 1.0.

**Table 3:** League table of all pairwise treatment comparisons for PASI 90 at week 16 Odds ratios (95% confidence intervals) for all pairwise comparisons. Read as row treatment versus column treatment; OR >1.0 favors row treatment. Bold values indicate direct head-to-head trial evidence. TNF-α: tumor necrosis factor-alpha; IL: interleukin.

	Adalimumab	Bimekizumab	Guselkumab	Placebo	Risankizumab	Secukinumab	Ustekinumab
Adalimumab (TNF-α)	—	0.18 [0.13–0.25]	0.37 [0.29–0.46]	30.07 [19.16–47.18]	0.32 [0.24–0.42]	0.40 [0.28–0.57]	1.13 [0.80–1.58]
Bimekizumab (IL-17A/F)	5.76 [3.73–8.89]	—	2.07 [1.40–3.05]	170.04 [104.33–277.14]	1.78 [1.30–2.45]	2.27 [1.71–3.01]	6.37 [4.77–8.52]
Guselkumab (IL-23p19)	2.77 [1.97–3.90]	0.48 [0.33–0.71]	—	82.25 [51.23–132.03]	0.86 [0.61–1.23]	1.10 [0.73–1.66]	3.08 [2.07–4.59]
Placebo	0.03 [0.02–0.05]	0.01 [0.01–0.01]	0.01 [0.01–0.02]	—	0.01 [0.01–0.02]	0.01 [0.01–0.02]	0.04 [0.02–0.06]
Risankizumab (IL-23p19)	3.17 [2.39–4.21]	0.56 [0.41–0.77]	1.16 [0.81–1.64]	95.40 [59.97–151.74]	—	1.27 [0.94–1.71]	3.58 [2.74–4.67]
Secukinumab (IL-17A)	2.49 [1.75–3.56]	0.44 [0.33–0.58]	0.91 [0.60–1.37]	75.01 [45.37–124.02]	0.79 [0.58–1.06]	—	2.81 [2.17–3.65]
Ustekinumab (IL-12/23)	0.89 [0.63–1.25]	0.16 [0.12–0.21]	0.32 [0.22–0.48]	26.68 [16.48–43.20]	0.28 [0.21–0.36]	0.36 [0.27–0.46]	—

Notable findings from the comprehensive pairwise comparisons include the following. Bimekizumab demonstrated statistical superiority over all other active treatments, with all ORs greater than 1.0 and confidence intervals excluding 1.0 when bimekizumab is in the row position. Risankizumab and guselkumab showed comparable efficacy to each other (OR 1.16, 95% CI 0.81-1.64). Secukinumab showed similar efficacy to both risankizumab and guselkumab. All newer biologic agents (bimekizumab, IL-23 inhibitors, and secukinumab) demonstrated substantial superiority over adalimumab and ustekinumab. Adalimumab and ustekinumab demonstrated similar efficacy to each other (OR 1.13, 95% CI 0.80-1.58).

Heterogeneity and Inconsistency

The overall network demonstrated very low heterogeneity: between-study variance (τ²) = 0.0000; global network heterogeneity (I²) = 0.0%; 95% CI for between-study variance: 0.0-60.2%. These findings indicate excellent consistency across the included studies. We observed moderate statistical heterogeneity for a single comparison: bimekizumab versus placebo (I² = 67.1%). This heterogeneity was observed between the two placebo-controlled bimekizumab trials (BE READY and BE VIVID). The moderate heterogeneity likely reflects differences in study populations: BE READY enrolled predominantly biologic-naive patients (87% with no prior biologic exposure) and was a two-arm placebo-controlled withdrawal design, while BE VIVID included a higher proportion of treatment-experienced patients (33% prior biologic exposure) in a three-arm design comparing bimekizumab, ustekinumab, and placebo. All other pairwise comparisons showed minimal heterogeneity (I² less than 1%).

We performed a comprehensive inconsistency analysis using the SIDE approach with the back-calculation method. All treatment comparisons demonstrated consistency between direct and indirect evidence, with no statistically significant disagreement detected (all p-values greater than 0.05). For the bimekizumab versus placebo comparison specifically, the agreement was excellent (RoR = 1.03, p = 0.96), indicating that direct and indirect evidence are in near-perfect alignment. Table 4 presents the complete inconsistency analysis results.

**Table 4:** Consistency analysis comparing direct versus indirect evidence SIDE analysis with back-calculation method. k: number of studies with direct evidence; Direct Prop.: proportion of information from direct evidence; RoR: Ratio of Ratios (direct OR/indirect OR); p-values >0.05 indicate no significant inconsistency between direct and indirect evidence.

Comparison	k	Direct Prop.	Network OR	Direct OR	Indirect OR	RoR	p-value	Interpretation
Bimekizumab vs. Placebo	2	0.28	170.04	173.34	168.80	1.03	0.96	Excellent
Bimekizumab vs. Adalimumab	1	0.54	5.76	7.00	4.41	0.63	0.17	Good
Bimekizumab vs. Secukinumab	1	0.58	2.27	2.04	2.62	0.78	0.39	Good
Bimekizumab vs. Ustekinumab	1	0.45	6.37	5.76	6.92	0.83	0.54	Good
Risankizumab vs. Secukinumab	1	0.39	1.27	1.47	1.16	1.27	0.44	Good
Risankizumab vs. Ustekinumab	2	0.63	3.58	3.72	3.34	1.12	0.70	Excellent
Risankizumab vs. Adalimumab	1	0.70	3.17	2.92	3.85	1.32	0.38	Good
Risankizumab vs. Placebo	2	0.35	95.40	77.21	107.02	0.72	0.51	Good
Guselkumab vs. Adalimumab	2	0.98	2.77	2.71	4.48	1.65	0.54	Good
Guselkumab vs. Placebo	2	0.59	82.25	93.32	68.75	1.36	0.53	Good
Secukinumab vs. Ustekinumab	1	0.58	2.81	2.77	2.86	0.97	0.91	Excellent
Adalimumab vs. Placebo	2	0.52	30.07	34.48	25.85	1.33	0.53	Good
Ustekinumab vs. Placebo	3	0.56	26.68	19.82	38.99	0.51	0.17	Good

Sensitivity Analyses

Results remained highly consistent across both fixed-effect and random-effects modeling approaches, with overlapping confidence intervals and similar point estimates. This indicates that the choice of statistical model did not materially affect our conclusions. The moderate heterogeneity in the bimekizumab versus placebo comparison (I² = 67.1%) can be explained by clinically meaningful differences between the two trials regarding patient populations and trial designs. Importantly, both trials demonstrated very large effect sizes strongly favoring bimekizumab over placebo (both contributing to a pooled OR greater than 170), differing only in the precise magnitude of the effect rather than the direction or clinical significance of the finding. Bimekizumab demonstrated excellent consistency across all three active comparator trials, with minimal heterogeneity (I² less than 1% for all comparisons versus adalimumab, secukinumab, and ustekinumab).

Publication Bias Assessment

**Figure 4:**
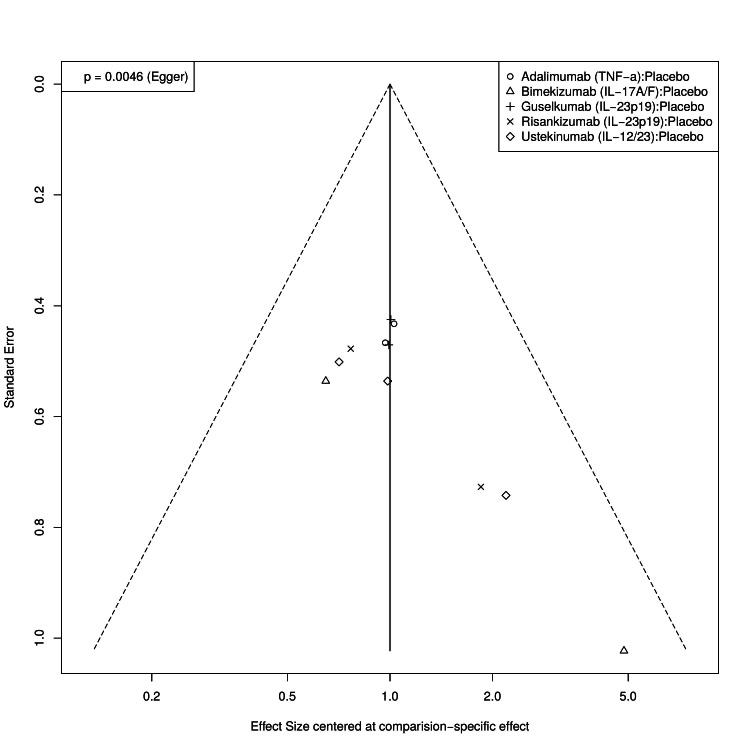
Funnel plot for assessment of publication bias Funnel plot displaying standard error versus log odds ratio for all pairwise treatment comparisons. Egger’s test (p = 0.0046) suggested potential small-study effects, while Begg-Mazumdar (p = 0.39) and Thompson-Sharp (p = 0.10) tests were not statistically significant.

Funnel plot asymmetry testing produced mixed results: Egger's regression test (p = 0.0046) suggested potential small-study effects, while the Begg-Mazumdar rank correlation test (p = 0.39) and Thompson-Sharp test (p = 0.10) did not reach statistical significance. Visual inspection of the funnel plot (Figure 4, Appendices) revealed slight asymmetry, with some smaller studies reporting larger treatment effect estimates. However, two of the three statistical tests did not reach statistical significance, suggesting that any potential publication bias is unlikely to substantially influence our conclusions.

Treatment Ranking

Based on OR compared to placebo, the efficacy hierarchy was as follows: bimekizumab (OR 170.04, 95% CI 104.33-277.14), risankizumab (OR 95.40, 95% CI 59.97-151.74), guselkumab (OR 82.25, 95% CI 51.23-132.03), secukinumab (OR 75.01, 95% CI 45.37-124.02), adalimumab (OR 30.07, 95% CI 19.16-47.18), and ustekinumab (OR 26.68, 95% CI 16.48-43.20). Bimekizumab demonstrated statistically significant superiority compared to all other active comparators (all pairwise comparisons favored bimekizumab with a 95% CI excluding 1.0). Risankizumab, guselkumab, and secukinumab formed a cluster of similarly effective treatments, with overlapping confidence intervals when compared to each other, but all showed clear superiority over adalimumab and ustekinumab.

Discussion

Principal Findings

This systematic review and NMA provide robust comparative effectiveness evidence for biologic therapies in moderate-to-severe plaque psoriasis. Our findings establish a clear efficacy hierarchy, with bimekizumab achieving the highest PASI 90 response rates, followed by risankizumab, guselkumab, and secukinumab, with all of these agents substantially outperforming adalimumab and ustekinumab.

Bimekizumab, which uniquely inhibits both IL-17A and IL-17F cytokines, achieved the highest efficacy, with 91% of patients reaching PASI 90 at week 16 in the placebo-controlled BE READY trial [11]. The drug demonstrated significant superiority over adalimumab (OR 5.65, 95% CI 4.08-7.84) and secukinumab (OR 2.27, 95% CI 1.71-3.01) and estimated superiority over both risankizumab (OR 1.78) and guselkumab (OR 2.07) based on network estimates. The enhanced efficacy of dual IL-17A/F inhibition may be mechanistically explained by independent contributions of IL-17F to psoriatic skin inflammation [23], as IL-17F shares structural and functional similarities with IL-17A and can independently promote keratinocyte activation and neutrophil recruitment.

Risankizumab and guselkumab demonstrated similar efficacy to each other (OR 1.16), and both showed substantial superiority over adalimumab and ustekinumab, which demonstrated significantly lower PASI 90 response rates of approximately 47-58% compared to the newer agents. These medications offer the additional practical advantage of less frequent dosing intervals (every 12 weeks for risankizumab and every eight weeks for guselkumab following induction) compared to bimekizumab and secukinumab, which require monthly or more frequent administration, which may translate into improved treatment adherence and higher patient satisfaction in real-world practice.

Despite targeting only IL-17A, secukinumab achieved robust efficacy with 79% of patients reaching PASI 90 in the CLEAR trial [18], clearly outperforming both adalimumab (OR 2.49, 95% CI 1.75-3.56) and ustekinumab (OR 2.81, 95% CI 2.17-3.65). The difference between bimekizumab and secukinumab (OR 2.27, 95% CI 1.71-3.01) was both statistically significant and clinically meaningful, with direct head-to-head evidence from the BE RADIANT trial [9] (OR 2.04, 95% CI 1.41-2.96) closely aligning with the network estimate.

Interestingly, adalimumab and ustekinumab showed similar efficacy to each other (OR 1.13, 95% CI 0.80-1.58), suggesting that within the category of traditional biologics, there is no clear efficacy advantage of one mechanism over the other.

Network Quality and Consistency

A major strength of this analysis is the exceptionally low heterogeneity observed across the entire treatment network (I² = 0.0%, τ² = 0.0000), indicating remarkable consistency between direct evidence from head-to-head trials and indirect evidence derived through network connections. This high degree of consistency strengthens our confidence in the network-derived estimates for treatment comparisons lacking direct head-to-head trial evidence. Our comprehensive inconsistency analysis using the SIDE approach revealed no significant disagreement between direct and indirect evidence for any treatment comparison in the network (all p-values greater than 0.05). Despite moderate statistical heterogeneity in the bimekizumab versus placebo comparison (I² = 67.1%), no true inconsistency was detected (p = 0.96, RoR = 1.03), with direct and indirect evidence in excellent agreement. The observed heterogeneity reflects between-study variability in effect magnitude due to differences in patient populations and trial designs, rather than fundamental disagreement between evidence pathways.

Clinical Implications

These findings carry several important implications for clinical practice. For treatment-naive patients with moderate-to-severe plaque psoriasis seeking high levels of skin clearance, bimekizumab appears to offer the highest probability of achieving a PASI 90 response. However, risankizumab and guselkumab provide excellent alternatives with the practical advantage of less frequent dosing requirements, which may be preferred by patients valuing convenience. Patients experiencing inadequate therapeutic response to adalimumab or ustekinumab should be considered for switching to bimekizumab, secukinumab, risankizumab, or guselkumab, which demonstrate substantially higher efficacy in our analysis.

While bimekizumab shows the highest efficacy in our analysis, treatment selection should be individualized based on multiple patient-specific factors. These factors include patient preferences regarding dosing frequency; differences in safety profiles between drugs (such as higher rates of oral candidiasis associated with bimekizumab compared to other biologic therapies, including risankizumab and guselkumab [8-10]), access considerations and cost-effectiveness; previous treatment history; and the presence of comorbidities such as inflammatory bowel disease (where IL-17 inhibition may be contraindicated) or psoriatic arthritis.

Comparison With Previous Meta-Analyses

Our findings show general consistency with previous network meta-analyses [6,7] while providing updated evidence that incorporates bimekizumab, which was not included in earlier comparative analyses. The 2021 Cochrane NMA by Sbidian and colleagues [6] found that IL-17 and IL-23 pathway inhibitors were superior to TNF-α inhibitors for achieving high levels of skin clearance, consistent with our results. Our study extends this previous work by including bimekizumab and demonstrating its superiority over secukinumab, leveraging recent head-to-head trials (BE RADIANT [9] and IMMerge [12]) that provide valuable direct evidence, and conducting comprehensive inconsistency analyses demonstrating excellent agreement between direct and indirect evidence.

Limitations

Our study has several limitations that should be acknowledged. First, we focused exclusively on short-term efficacy (PASI 90 at week 16). Long-term efficacy, durability of response, and maintenance of remission are important clinical outcomes that were not assessed in this analysis. The relative ranking of treatments may differ at later timepoints. 

Second, we did not conduct a formal comparative safety analysis. Safety profiles differ meaningfully among biologic agents, and treatment selection should consider both efficacy and safety data. Notably, bimekizumab is associated with higher rates of oral candidiasis compared to other agents, which is an important consideration in clinical decision-making. Heterogeneous reporting of adverse events across trials precluded a formal comparative safety analysis. 

Third, all included trials were industry-sponsored, which may introduce potential bias. However, all trials demonstrated low risk of bias using the Cochrane RoB 2 tool [15], and the consistency of findings across multiple independent studies and sponsors provides reassurance. 

Fourth, the observed heterogeneity in the bimekizumab versus placebo comparison (I² = 67.1%), while clinically explainable and not representing true inconsistency, introduces some uncertainty in the precise magnitude of bimekizumab’s effect compared to placebo. However, this does not affect comparisons with active treatments, which showed excellent consistency. 

Fifth, our search was limited to two databases (PubMed and Clinical Trials). While these databases provide comprehensive coverage of published phase III trials, additional sources such as Embase or the Cochrane Central Register of Controlled Trials might have identified additional records. 

Sixth, our analysis employed odds ratios versus placebo for treatment ranking alongside P-scores. While SUCRA provides probabilistic ranking with uncertainty quantification, P-scores derived from the frequentist netmeta package provide an equivalent ranking metric and are reported here alongside odds ratios to facilitate comprehensive treatment comparison.

## Conclusions

This systematic review and network meta-analysis demonstrate that bimekizumab achieves the highest efficacy for PASI 90 response at week 16 among the biologic therapies evaluated. Risankizumab, guselkumab, and secukinumab form a cluster of highly effective treatments with comparable efficacy, all demonstrating substantial superiority over adalimumab and ustekinumab. These findings can inform evidence-based treatment selection, guideline development, and shared decision-making for patients with moderate-to-severe plaque psoriasis. Treatment choice should integrate efficacy data with considerations of dosing convenience, safety profiles, patient preferences, and individual clinical circumstances.

## Appendices

**Table 5:** Detailed risk of bias assessment using the Cochrane RoB 2 tool Risk of bias assessment for all 11 included trials using the Cochrane Risk of Bias 2 tool across five domains: randomization process, deviations from intended interventions, missing outcome data, measurement of the outcome, and selection of the reported result. Assessment was performed independently by two reviewers, with disagreements resolved through discussion. All trials demonstrated low risk of bias across all domains.

Study	D1: Randomization	D2: Deviations	D3: Missing Data	D4: Measurement	D5: Reported Result	Overall
BE SURE (Warren 2021)	Low	Low	Low	Low	Low	Low
BE RADIANT (Reich 2021)	Low	Low	Low	Low	Low	Low
BE VIVID (Reich 2021)	Low	Low	Low	Low	Low	Low
BE READY (Gordon 2021)	Low	Low	Low	Low	Low	Low
CLEAR (Langley 2015)	Low	Low	Low	Low	Low	Low
VOYAGE-1 (Blauvelt 2017)	Low	Low	Low	Low	Low	Low
VOYAGE-2 (Reich 2017)	Low	Low	Low	Low	Low	Low
IMMerge (Warren 2021)	Low	Low	Low	Low	Low	Low
UltIMMa-1 (Gordon 2018)	Low	Low	Low	Low	Low	Low
UltIMMa-2 (Gordon 2018)	Low	Low	Low	Low	Low	Low
IMMvent (Reich 2019)	Low	Low	Low	Low	Low	Low
